# Comparative analysis of four nuclei in the human brainstem: Individual differences, left-right asymmetry, species differences

**DOI:** 10.3389/fnana.2023.1069210

**Published:** 2023-02-16

**Authors:** Joan S. Baizer, Sandra F. Witelson

**Affiliations:** ^1^Department of Physiology and Biophysics, Jacobs School of Medicine and Biomedical Sciences, University at Buffalo, Buffalo, NY, United States; ^2^Department of Psychiatry and Behavioural Neurosciences, Michael G. DeGroote School of Medicine, Faculty of Health Sciences, McMaster University, Hamilton, ON, Canada

**Keywords:** cerebellum, inferior olive, dorsal cochlear nucleus, arcuate nucleus of the medulla, left-right asymmetry, nucleus paramedianus dorsalis

## Abstract

**Introduction:**

It is commonly thought that while the organization of the cerebral cortex changes dramatically over evolution, the organization of the brainstem is conserved across species. It is further assumed that, as in other species, brainstem organization is similar from one human to the next. We will review our data on four human brainstem nuclei that suggest that both ideas may need modification.

**Methods:**

We have studied the neuroanatomical and neurochemical organization of the nucleus paramedianus dorsalis (PMD), the principal nucleus of the inferior olive (IOpr), the arcuate nucleus of the medulla (Arc) and the dorsal cochlear nucleus (DC). We compared these human brainstem nuclei to nuclei in other mammals including chimpanzees, monkeys, cats and rodents. We studied human cases from the Witelson Normal Brain collection using Nissl and immunostained sections, and examined archival Nissl and immunostained sections from other species.

**Results:**

We found significant individual variability in the size and shape of brainstem structures among humans. There is left-right asymmetry in the size and appearance of nuclei, dramatically so in the IOpr and Arc. In humans there are nuclei, e.g., the PMD and the Arc, not seen in several other species. In addition, there are brainstem structures that are conserved across species but show major expansion in humans, e.g., the IOpr. Finally, there are nuclei, e.g. the DC, that show major differences in structure among species.

**Discussion:**

Overall, the results suggest several principles of human brainstem organization that distinguish humans from other species. Studying the functional correlates of, and the genetic contributions to, these brainstem characteristics are important future research directions.

## Introduction

Since the pioneering neuroanatomical studies of Ramón y Cajal (Ramón y Cajal, 1995), neuroanatomical studies have used a number of mammalian species, most commonly cats, rabbits, rats and mice. Relatively fewer studies have used human tissue. One of the tools relied on by neuroanatomists is the comparison of sections processed in individual laboratories with “standard” sections as illustrated in various published brain atlases (some examples: Berman, 1968, cat brainstem; Franklin and Paxinos, 1997, mouse brain; Paxinos, 1999, rat brainstem; Paxinos et al., 2000, monkey brain; Paxinos and Watson, 2007, rat brain). The use of these atlases is based on the premise that the brains from any given species are very similar one to the next, i.e., that a section at a given plane and level from an individual of a given species will very closely resemble sections from other individuals of that species. In human cortex, individual variability of sulcal and gyral configurations has posed a major problem in the construction of atlases (discussion in Van Essen et al., 1998). However, there are atlases of human brainstems (Olszewski and Baxter, 1982; Büttner-Ennever et al., 2014), again implying similarity of brainstem structures across individuals.

In this review we will consider and synthesize data on the neuroanatomy and neurochemistry of four nuclei in the human brainstem from our earlier studies, and compare the organization of the human brainstem to that in other species. Our analysis suggests five major concepts about human brainstem organization. First, there is significant individual variability in the organization of brainstem structures in humans. Second, there is left-right asymmetry in several brainstem structures in humans. Third, there are brainstem nuclei found in humans but not in other species. Fourth, there are brainstem structures that are conserved across species but which show major expansion in humans. Fifth there are brainstem structures that show major differences in organization among species.

## Materials and methods

### Human cases

We studied human brainstems from the Witelson Normal Brain Collection (WNBC), only strongly right-handed cases were selected (Witelson and McCulloch, 1991), as well as small blocks of brainstem from the Ichan School of Medicine at Mt. Sinai (ISMMS). Table 1 shows critical parameters of the cases including case number, age, sex, and post-mortem interval (PMI, in hours).

**TABLE 1:** Human cases.

Case	Source	Age	Sex	PMI (h)
120	WNBC	25	m	4
125	WNBC	57	m	5
150	WNBC	63	m	3
155	WNBC	50	f	9
158	WNBC	51	m	1
164	WNBC	45	f	3
166	WNBC	65	f	3
167	WNBC	55	f	2
168	WNBC	69	m	3
169	WNBC	70	m	2
171	WNBC	63	f	6
176	WNBC	71	f	3
180	WNBC	54	m	2
1,342	ISMMS	36	m	17
1,130	ISMMS	38	m	24
1,057	ISMMS	40	f	4
1,319	ISMMS	48	m	17

### Histological procedures: Human tissue

The details of tissue acquisition for the Witelson Normal Brain Collection were given in Witelson and McCulloch (1991). Our methods for processing this tissue have been described previously (Baizer et al., 2007). Briefly, the brains were stored in 10% formalin. The brainstems were dissected away from the cerebrum and cerebellum and then cryoprotected in 15% then 30% sucrose in 10% formalin. Prior to sectioning, we made a small slit along one side of the ventral brainstem to allow identification of left and right sides of the brain. Forty μm thick frozen sections were cut on an American Optical sliding microtome in a plane transverse to the brainstem. All sections were collected and stored in plastic compartment boxes, 5 sections/compartment, at 4°C in 5% formalin. Initially, sets of sections 2 mm apart from each case were stained with a Nissl stain, Cresyl Violet (CV), following a standard protocol (LaBossiere and Glickstein, 1976). Additional sections were CV-stained as needed to define the limits of structures of interest. We also sectioned and stained small blocks of tissue containing the inferior olive from ISMMS.

### Histological procedures: Animal tissue

For this review we also examined and photographed archival slides of brainstems from chimpanzees, monkeys, cats and several rodents (chinchilla, rat, guinea pig). Table 2 lists the animal cases. Cresyl and immunostained sections from several species (chimpanzee, monkey, cat, chinchilla, guinea pig, rat) were prepared in the laboratory at the University at Buffalo (Baizer and Baker, 2005, 2006a,b; Manohar et al., 2012; Baizer et al., 2013a,^2015^; Paolone et al., 2014). Details of tissue acquisition and histology were given in those publications. In addition, we examined stained sections of chimpanzee, monkey and cat brains that had been prepared in other laboratories (see Table 2).

**TABLE 2:** Animal cases.

Species	Source	*n*
**Processed in Buffalo**		
Cat	James F. Baker, Northwestern University	10
Chimpanzee	Chet Sherwood, George Washington University Patrick Hof, Icahn School of Medicine at Mount Sinai	3
Chinchilla	Richard Salvi, University at Buffalo	2
Guinea pig	Susan Shore, University of Michigan	2
Macaque monkey	David B. Bender, University at Buffalo Chet Sherwood	3
Rat	Richard Salvi	25
Squirrel monkey	James F. Baker	5
**Slides from other laboratories**		
Cat	Mitchell Glickstein, Brown University	1
Macaque monkey	Mitchell Glickstein	1
Chimpanzee	Chet Sherwood	5

### Antibodies and immunohistochemistry (IHC)

All IHC was done on free-floating sections. Sections were rinsed in PBS (all rinses were 3 × 10 min) and then treated with an antigen retrieval (AR) protocol. Each section was placed in a separate small glass jar with 20 ml of pH = 6 citrate buffer. The jars were heated in a water bath at 85°C for 30 min. The jars were removed from the bath and cooled to room temperature. Next, sections were rinsed in PBS and then non-specific label was blocked by incubating sections in a solution of phosphate buffered saline (PBS), 0.1-1% Triton-X 100,1% bovine serum albumin and 1.5% normal serum. The primary antibody was added and sections incubated with the overnight at 4°C on a tissue rocker. Further processing was with the Vector “ABC” method using the appropriate Vector Elite kit (Vector Laboratories, Burlingame, CA) followed by visualization with a 3,3’-diaminobenzidine (Sigma-Aldrich, now Thermo Fisher) protocol, giving brown staining, or a glucose-oxidase modification of the protocol giving gray-black staining (Shu et al., 1988; Van Der Gucht et al., 2006). Sections were mounted on gelled or electrostatically charged slides, dehydrated in 70, 95, and 100% alcohol, cleared in Xylene and coverslipped with Permount (Fisher Scientific). Table 3 summarizes the primary antibodies and dilutions used in these studies. Details of antibody specificity and controls were given in the individual publications.

**TABLE 3:** Primary antibodies and dilutions.

Antigen	Manufacturer, Cat. #	Host	Dilution
Calbindin (CB)	Chemicon, AB1778	Rb	1:2,000
Calretinin (CR)	Chemicon, AB1568	Ms	1:1,000
Calretinin (CR)	Chemicon, AB5054	Rb	1:2,000–1:3,000
Glutamic acid decarboxylase (GAD_65/67_)	Chemicon/Millipore, AB1511	Rb	1:1,000
Nitric oxide synthase (nNOS)	Cayman, 160870	Rb	1:200
Nonphosphorylated neurofilament protein (NPNFP)	Covance, SMI-32	Ms	1:1,000
Parvalbumin (PV)	Sigma, P3088	Ms	1:2,000

Sections from perfused animal brains were processed in the same way except that the AR procedure was omitted.

### Data analysis and photography

We examined sections with a Leitz Dialux 20 light microscope. We and captured digital images (1,600 × 1,200 pixels) with either a SPOT Insight Color Mosaic camera or a Wild Makroskope mounted on the Leitz microscope. Brightness, contrast and color of the images were adjusted and figures assembled with Adobe Photoshop software (San Jose, CA). Some of the slides used in the photomicrographs for this paper had been photographed in earlier reports.

### Overall strategy

We used Nissl-stained sections to assess the size and shape of different structures. We also used immunohistochemistry to examine the expression of several proteins in brainstem nuclei. The selection of antibodies was based on prior work in cerebral cortex and thalamus. We asked two major questions: first were there neurochemically-defined subdivisions of any nuclei? Earlier studies had found expression of different calcium-binding proteins distinguished anterolateral and dorsal column system afferents in thalamus (Rausell et al., 1992). We had found that expression of calcium-binding proteins showed subdivisions in the vestibular nuclear complex of several species (Baizer and Baker, 2005, 2006a,b). The second question was whether there were distinct neurochemically-defined cell populations in brainstem nuclei as had been seen for cortical structures. Subpopulations of interneurons immunoreactive for different calcium-binding proteins had been described in cortex (DeFelipe et al., 1989; Glezer et al., 1993; Condé et al., 1994). Expression of non-phosphorylated neurofilament protein (NPNFP) labeled subpopulations of cortical pyramidal cells (Hof et al., 1990, 1996; Hayes and Lewis, 1992; Hof and Morrison, 1995).

## Results

We will summarize our analysis of four different nuclei in the human brainstem; the results of those studies led to the key concepts of human brainstem organization that we are developing here.

### Brainstem structures found in human but not other species

**FIGURE 1:**
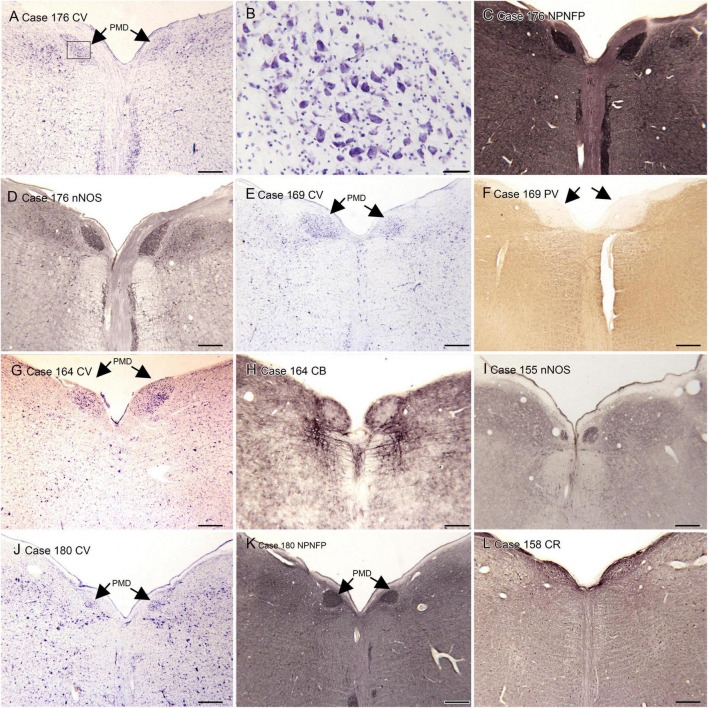
The nucleus paramedianus dorsalis (PMD) in the human brain. **(A)** The arrows show the location of PMD on a CV-stained section from Case 176. The rectangle shows the location of the higher magnification image in panel **(B)**. **(B)** Neurons in the PMD are large and multipolar. **(C)** Immunoreactivity for non-phosphorylated neurofilament protein (NPNFP) in PMD; the nucleus is darkly labeled. Note the difference in shape between left and right sides. **(D)** nNOS expression in PMD. **(E)** The arrows indicate PMD in Case 169 shown on a CV-stained section. Note that it is larger on the left than on the right. **(F)** The arrows indicate PMD; note the absence of immunoreactivity to PV of neurons in PMD. **(G)** The arrows indicate PMD in Case 164. There is a slight difference in shape between left and right sides. **(H)** Neurons in PMD do not express the calcium-binding protein calbindin (CB). **(I)** Expression of nNOS in PMD of Case 155. **(J)** PMD in Case 180; in this case the nucleus is smaller and almost round. **(K)** The neurons and processes in PMD express non-phosphorylated neurofilament protein; the shape of the nucleus is much easier to see in the immunostained section. **(L)** Neurons in PMD express the calcium-biding protein calretinin (CR). Scale bars: **(A,C–L)** = 500 μm; **(B)** = 50 μm.

**FIGURE 2:**
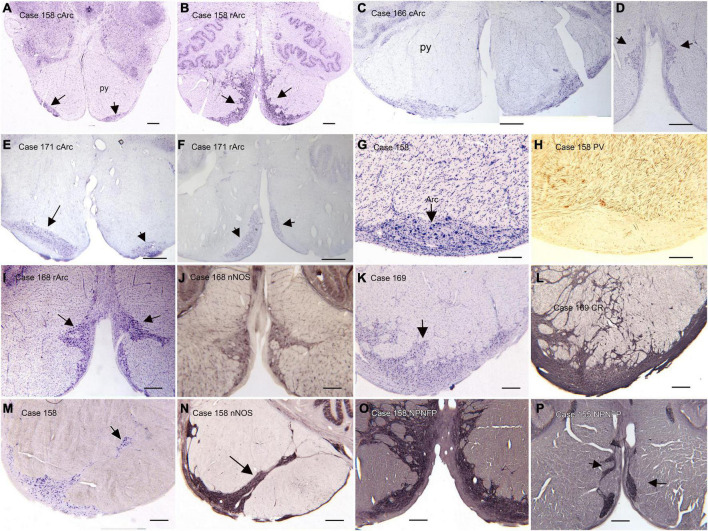
The Arcuate nucleus (Arc) of the medulla. There are two distinct subdivisions, the caudal (cArc) and rostral (rArc) subdivisions. **(A)** The cArc (arrows) on a CV-stained section from Case 158. The cArc is ventrolateral to the pyramidal tracts (py) at the ventral surface of the brainstem. **(B)** The rArc (arrows) in Case 158. The rArc is ventromedial to the py and extends up the midline. The section in panel **(B)** is 14 mm rostral to the one in panel **(A)**. Note the left-right asymmetry in panels **(A,B)**. **(C,D)** cArc and rArc in Case 183. **(E)** cArc (arrows) in Case 171. In this case, the Arc on the left is dorsal to the surface of the brainstem, embedded in the py. **(F)** rArc in Case 171 on a section 10 mm rostral to the one in panel **(E)**. **(G)** The Arc on a CV-stained section adjacent to a section **(H)** immunostained for parvalbumin (PV). **(I)** The Arc on a CV-stained section adjacent to a section **(J)** immunostained for nitric oxide synthase (nNOS). **(K)** The Arc on a CV-stained section adjacent to a section **(L)** immunostained for calretinin (CR). Arc neurons and processes express CR. **(M)** The Arc on a CV-stained section. **(N)** Adjacent section immunostained for nNOS. In this case the Arc extends well into the py. **(O,P)** Sections from two different cases immunostained for non-phosphorylated neurofilament protein (NPNFP). Neurons and processes express NPNFP, and the Arc stands out from surrounding white matter of the py. Scale bars: **(A–F)** = 1 mm; **(I–P)** = 500 μm; **(G,H)** = 250 μm.

We will describe two examples. The first is the nucleus paramedianus dorsalis, PMD, and the second is the arcuate nucleus of the medulla, Arc (Baizer et al., 2007, 2022). Individual variability was seen in both; the arcuate also shows left-right asymmetry.

(A) The nucleus paramedianus dorsalis (PMD) is a small nucleus found in the medulla. This nucleus is identified in the human atlas of Olszewski and Baxter (1954) (plates XIV- XIX, caudal and oral subdivisions). In the atlas of (Paxinos and Huang, 1995) the caudal subdivision of the nucleus is shown from Obex + 9 to Obex + 12 (labeled CDPMn, caudal dorsal paramedian nucleus), not at Obex 13 and the oral subdivision at Obex14 (labeled ODPMn, oral dorsal paramedian nucleus). No such nucleus is shown in atlases of other species, including the cat, the monkey and rat (Berman, 1968; Paxinos, 1999; Paxinos et al., 2000). We analyzed its rostro-caudal extent of PMD in 8 cases using Nissl and immunostained sections and found a range of 4–7 mm. We also found individual differences in PMD shape and size. Figure 1 shows PMD on cresyl-violet and immunostained sections from Case 155 (Figure 1I), 158 (Figure 1L), Case 164 (Figures 1G–I), Case 169 (Figures 1E, F), Case 176 (Figures 1A–C) and Case 180 (Figures 1J–L). The neurons of PMD are large, typically multipolar neurons (Figure 1B). The neurons of PMD express nitric oxide synthase (nNOS, Figures 1D, I) calretinin (CR Figure 1L) and non-phosphorylated neurofilament protein (NPNFP, Figures 1C, K, L) and not calbindin (CB, Figure 1H) or parvalbumin (PV, Figure 1F). This figure also show the case-to-case variability in the size and shape of the nucleus. It is almost circular in Case 180 (Figures 1J–L) and elongated in Cases 176 (Figures 1A, C, D) and 158 (Figure 1L).

(B) The arcuate nucleus of the medulla (Arc) is shown in the atlas of Olszewski and Baxter (1954) and the atlas of Paxinos and Huang (1995), but its size and rostro-caudal extent are very different in the two atlases. In the atlas of Olszewski and Baxter (1954) the Arc is shown as discontinuous, present on plate VIII, (section 2301), plate X (section 2051), not on plate XII and again on plates XIV (section 1801), XVI (section 1701), and XVIII (section 1601), with a total rostro-caudal extent of 9 mm. However, in the Paxinos and Huang (1995) atlas it is present only at Obex + 2 and Obex + 3, for a rostro-caudal extent of about 2 mm. This discrepancy between atlases suggests that there may be major individual differences in the extent of the Arc. We compared the size and shape of the Arc in Nissl and immuno-stained sections from 13 human cases (Baizer et al., 2022). We found major variability among cases in the size and shape of the Arc at any given rostro-caudal level, as well as in its total rostro-caudal extent. In addition, we found left-right asymmetry in the size and position of the Arc in each case and at all rostro-caudal levels. In the majority of cases, the Arc could be divided into two subnuclei, a caudal Arc (cArc) and a rostral Arc (rArc). In some cases these were continuous, in others there was a gap between them and in still other cases both cArc and rArc were present on a single section. The atlas of Olszewski and Baxter (1954) illustrates both cArc and rArc, the atlas of Paxinos and Huang (1995) shows only the cArc. Figure 2 illustrates the major features of the Arc on Nissl and immuno-stained sections from 6 Cases (155, 158, 168, 169, 171, 183). Figures 2A–F show pairs of CV-stained sections from 3 cases illustrating the differences in both subdivisions. These images also illustrate the left-right asymmetry in the Arc. Asymmetry is relatively minor in Case 158 (Figures 2A, B) and more apparent in both Cases 183 and 171 (Figures 2C–F). In some cases, the neurons and processes of the Arc infiltrate the py, and the Arc is not confined to the surface of the medulla (Figures 2L–N). We also examined immunostained sections through the Arc. We illustrate the major findings on pairs of adjacent sections, one stained for CV and the second immunostained. Neurons in the Arc immunostained for nitric oxide synthase (nNOS, Figures 1J, N), calretinin (CR, Figure 2L) and non-phosphorylated neurofilament protein, (NPNFP, Figures 2O, P) and do not express parvalbumin (PV, Figure 2H).

### A brainstem structure that is conserved across species but shows major expansion in humans: The principal nucleus of the inferior olive, IOpr

**FIGURE 3:**
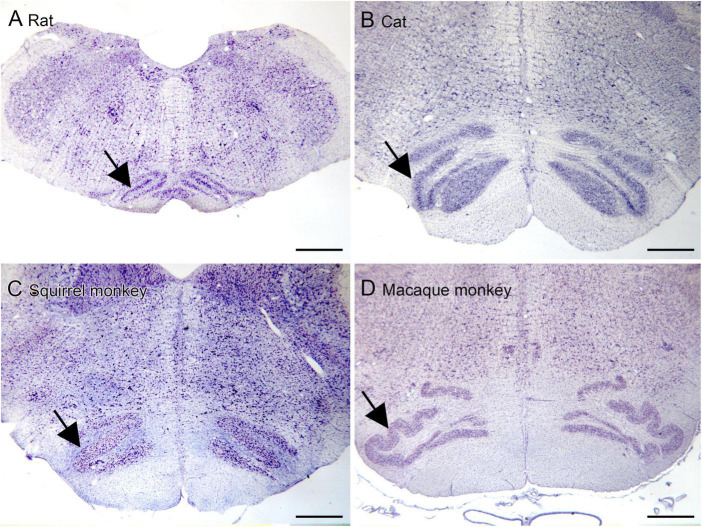
The IOpr (arrows) in rat **(A)**, cat **(B)**, squirrel monkey **(C)** and macaque monkey **(D)**. In the rat, cat and squirrel monkey the IOpr is a simple folded ribbon. In the macaque monkey, the folding pattern is more complex but the IOpr shows left-right symmetry. Scale bars = 1 mm.

**FIGURE 4:**
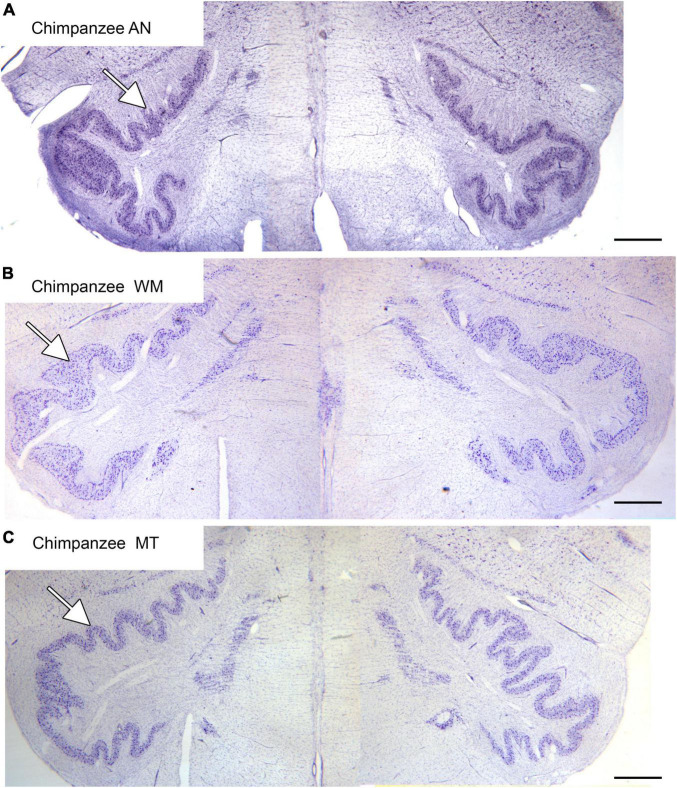
The IOpr in the chimpanzee. The IOpr (arrows) in three different chimpanzees, AN **(A)**, WM **(B)**, and MT **(C)**. The folding pattern is much more complex than in the species shown in Figure 3. Further, the shape and folding pattern of the IOpr is different for each animal, and there is left-right asymmetry all three animals. Scale bars: **(A–C)** = 1 mm.

**FIGURE 5:**
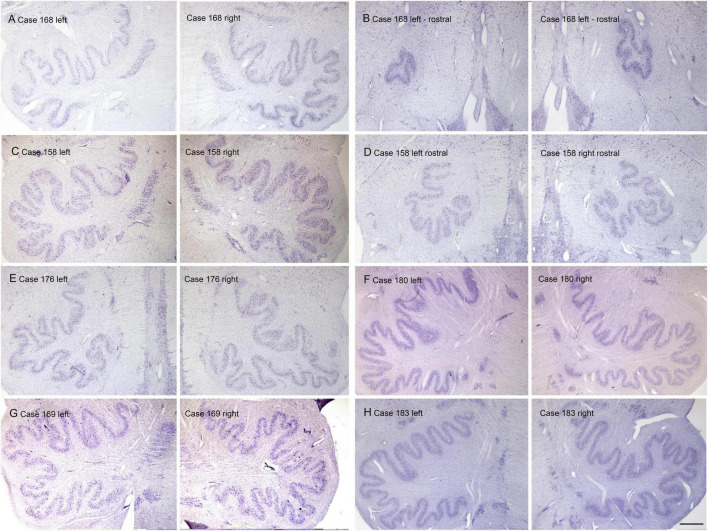
Individual variability and left-right asymmetry in the principal nucleus of the inferior olive (IOpr) in the human. Each panel **(A–H)** shows a pair of images from the left and right sides of the brain. For two cases 168 **(A,B)** and 158 **(C,D)** there are two panels showing pairs of sections at two different rostro-caudal levels. **(E)** Case 176. **(F)** Case 180. **(G)** Case 169. **(H)** Case 183. The asymmetry is present at all levels. Scale bar = 1 mm (all panels).

The inferior olive is the sole source of climbing fibers that innervate the cerebellum (Armstrong, 1974; Desclin, 1974; Voogd et al., 1990). While it is present in all mammals with a cerebellum, its size and organization vary dramatically among species (Kooy, 1917). The inferior olive is comprised of a number of subdivisions, including the dorsal, medial and principal (IOpr) nuclei (Olszewski and Baxter, 1954; Paxinos and Huang, 1995). Our studies have focused on the IOpr as it is this subdivision that varies dramatically in size and form among species. Figure 3 shows the IOpr in CV-stained sections from 4 species. The IOpr has the form of a band or ribbon. In the rat, cat and squirrel monkey (Figures 3A–C) this ribbon has one bend so that the IOpr is a “u-shaped” structure. In those three species it is bilaterally symmetrical. In the macaque monkey, the IOpr is a band with several infoldings (Figure 3D). However, the left and right sides show an almost identical folding pattern. Figure 4 shows the IOpr in the brains of three chimpanzees. In this species the IOpr ribbon has greatly expanded with a complex pattern of infoldings. The shape of the IOpr ribbon is different among the three cases. Further, for each case, there is a difference in the folding pattern between left and right sides. This expansion of the IOpr, individual differences in its shape and left-right asymmetry in all cases is seen much more dramatically in the human (Baizer et al., 2011, 2018). The extent of the IOpr expansion in the human is such that the whole ventral half of the medulla has expanded around it. In the human cases there are three major findings. There is individual variability both in the size and shape of the IOpr and in the folding pattern of the IOpr “ribbon.” For each case, and at every level examined, the folding pattern was different on left and right sides of the brain. All of these observations are illustrated in Figure 5 which shows the IOpr in Cases 168 (A, B; are from two different rostro-caudal levels), Case 158 (C, D, again from different rostro-caudal levels), Case 176 (E), Case 180 (F), Case 169 (G) and Case 183 (H).

**FIGURE 6:**
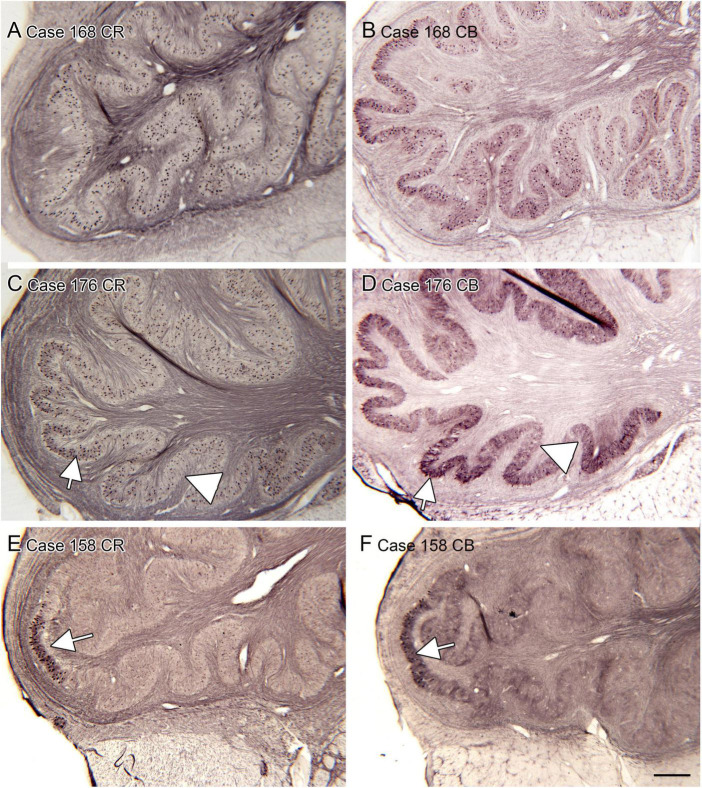
Variability in the expression of calcium-binding proteins calretinin (CR) and calbindin (CB) in the IOpr of humans. We show pairs of sections from three different cases showing CR-ir and CB-ir neurons. **(A,B)** Case 168; there are many immunolabeled neurons over the IO ribbon with some local differences in density. **(C,D)** Case 176. **(E,F)** Case 158. Note the (arrows) small patches of densely immunoreactive cells. Scale bar in panel **(F)** = 500 μm (applies to all panels).

We also asked if there might also be within or between individual differences in the neurochemistry of IOpr neurons. We found immunolabel for two calcium-binding proteins, calbindin (CB) and calretinin (CR) in IOpr neurons. However, there was variability both within and among cases in the number and distributions of neurons immunoreactive to those proteins. Figure 6 shows immunoreactivity for CR and CB in the IOpr of three different cases. In Case 168 there are scattered neurons throughout the IOpr immunolabeled for CR (Figure 6A) and CB (Figure 6B), suggesting that the two proteins are colocalized. In a second case, Case 176, there are also immunolabeled cells but these are not evenly distributed with areas of higher and lower densities of immunolabeled cells. In Figures 6C, D the arrows show regions with a higher density of labeled neurons, and the arrowheads show areas of lower density. The regional variation of the density of immunolabeled neurons is even more dramatic in Case 158 (Figures 6E, F). The arrows indicate a small extent of the IOpr in which neurons are densely immunolabeled surrounded by regions with only a few scattered immunolabeled cells. The variations in immunolabel for calcium-binding proteins, and the well-documented accumulation of lipofuscin in IOpr neurons (Moreno-Garcia et al., 2018) were consistent with the idea of a neurodegenerative process affecting the IOpr. To investigate this possibility we processed sections with a silver-staining kit (Baizer et al., 2018). We found that many IOpr neurons contain silver granules, and that there was an increase of such neurons with age.

### Species variability: The dorsal cochlear nucleus (DC)

**FIGURE 7:**
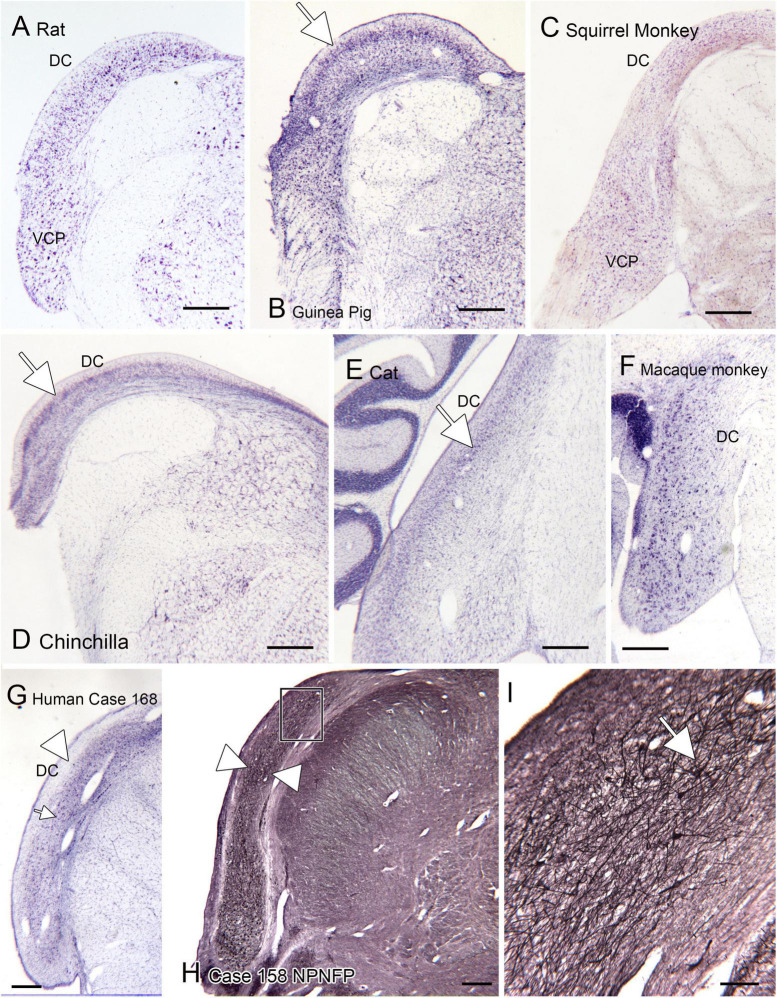
The dorsal cochlear nucleus (DC) in rat **(A)**, guinea pig **(B)**, squirrel monkey **(C)** and chinchilla **(D)**. **(E)** Cat. The arrow shows the layer of fusiform cells in the DC. **(F)** The DC in macaque monkey. **(G)** The DC in the human; CV-stained section. The arrowhead indicates an outer cell-sparse band about 200 μm thick. Deep to that band are scattered somata (example at arrowhead). **(H)** Expression of non-phosphorylated neurofilament protein (NPNFP) in the DC of the human. There is a broad band of immunolabel (between the arrowheads). The rectangle shows the location of the image in panel **(I)**. **(I)** The band of label is comprised of NPNFP-ir neurons (example at arrow) and a dense meshwork of immunostained processes. Scale bars: **(A–H)** = 500 μm; **(I)** = 100 μm.

One brainstem structure that shows marked variability among species is the dorsal cochlear nucleus of the medulla, the first relay of the auditory pathways. Figure 7 shows the DC in 7 different species. One of the first species studied was the cat (Brawer et al., 1974). Figure 7E shows a CV-stained section through the DC of the cat. In this species, the DC has a laminar organization, with a row of fusiform (also called pyramidal) neurons (arrow) that are aligned perpendicular to the surface. This DC organization established the idea that the DC is a laminar structure. However, a clear layer of pyramidal cells aligned perpendicular to the surface is not seen in all species. Figures 7A, B, D show the DC in three different rodents. The laminar organization is not striking in the rat (Figure 7A). In the chinchilla the most striking feature is an outer layer of granule cells (Figure 7D, arrow). There are large neurons in the macaque monkey (Figure 7F) but these are not as tightly aligned as in the cat. In a New World monkey, the squirrel monkey (Figure 7C) larger neurons are scattered but not clearly organized into a layer. Finally, Figures 7G–I show the DC in human. In a CV-stained section (Figure 7G) the outermost region is devoid of cells (arrowhead). There are scattered large neurons (example at arrow), but these are not aligned as in the cat and rodents. Immunoreactivity for NPNFP showed a broad band of label (Figure 7H, between the arrowheads) that included the region in which CV-stained somata were seen. This band was made up of a dense network of immunostained processes (Figure 7I) with scattered immunostained somata (Figure 7I, example at arrow). These somata were found over a range of depths and orientations within the broader band of immunoreactivity.

**FIGURE 8:**
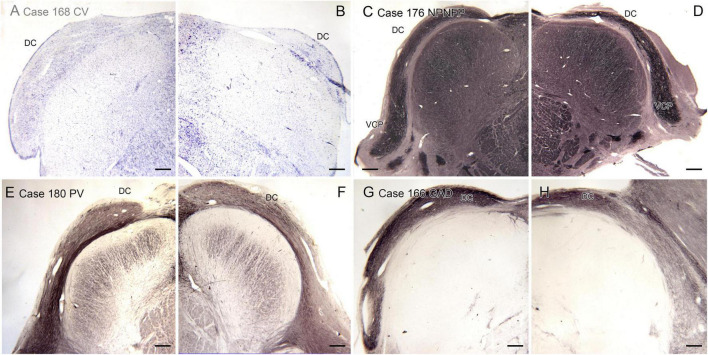
Differences in the rostro-caudal level of the DC on left and right sides of a single section. **(A,B)** The DC in Case 168, CV-stained section. The DC on the right is smaller than on the left, consistent with a more caudal level. **(C,D)** The DC and VCP on the left and right sides of Case 176, section immunostained for NPNFP. The DC is smaller on the right. **(E,F)** Case 180, section immunostained for PV. The DC on the left is smaller. **(G,H)** The DC in Case 166, section immunostained for GAD_65/67_. The left side is caudal to the right. Scale bars = 500 μm.

We also analyzed the possibility of left-right asymmetry in the DC. Figure 8 shows the left and right DC on single slides. In each case there is a small difference in the apparent rostro-caudal level of the DC between left and right sides. In Cases 168 and 176, the right side is caudal to the left, in Cases 166 and 180 the left side is caudal to the right. One interpretation of these images that there is a genuine left-right difference in the level of the appearance of the DC and that left and right sides are displaced relative to each other. However, it is also possible that these differences simply reflect an asymmetry in the cutting angle. The left and right DC at similar rostro-caudal levels do not show the dramatic differences in configuration between left and right sides seen for the IOpr and the Arc.

## Discussion

### The brainstem as the “reptilian brain”

We have found evidence for complexities in human brainstem organization including individual differences in structures, left-right asymmetry and nuclei not seen in other species. These observations are unexpected from the perspective of a persistent view of human brain organization derived from the writings of Paul MacClean (MacLean and Kral, 1973). They proposed that the brainstem, or “reptilian brain,” is conserved across species, and it is only the neocortex that expands and reaches its highest development in humans. Clearly, that view is in need of revision. First, as is apparent just from photographs of the human brain, the cerebellum expands in parallel with the cerebral cortex. Neuroanatomical data suggest that they function in parallel: the cerebellum receives input from the cerebral cortex via relays in the pontine nuclei (Brodal, 1968; Glickstein et al., 1972, 1985). The cerebellum in turn projects back to the cerebral cortex via thalamic relays (Middleton and Strick, 1997; Dum and Strick, 2003). The cerebellum and cerebral cortex thus communicate with each other only by way of relays in the brainstem and thalamus, suggesting the possibility that there might be parallel expansion of some of those relays.

### PMD and Arc: Human-specificity

Neither the connections nor the functions of the PMD have been studied. We previously suggested that it might be derived from the nucleus prepositus hypoglossi, and similarly play a role in eye movement control (Baizer et al., 2007). At present there is no way to test this hypothesis. Since PMD is absent in animal brains the classical ways of studying connections and function (tract tracing, electrophysiology, lesions-behavior) are not possible. The small size of PMD make applying functional and structural imaging techniques in humans likewise problematic, at least at the present time.

Individual variability in the human Arc has been described previously (Essick, 1912; Paradiso et al., 2018). Some authors consider it a precerebellar nucleus (Rasmussen and Peyton, 1946; Olszewski and Baxter, 1954). A different view of its function is derived from a clinical literature implicating the Arc in control of respiration and therefore as a candidate structure affected in Sudden Infant Death Syndrome (SIDS; Folkerth et al., 2008; Machaalani and Waters, 2008; Kinney, 2009; Ambrose et al., 2019). That view has been challenged (review and discussion in Baizer et al., 2022). While an Arc is a prominent feature in the human brain, we did not see evidence of this nucleus in other species including monkeys, cats, and rats and, nor is it depicted in atlases of those species (Berman, 1968; Paxinos and Watson, 1997; Paxinos et al., 2000). In the chimpanzee, we did find a well-developed Arc in one of eight cases, an Arc consisting of a few cells in another, and altogether absent in the other six cases (Baizer et al., 2013a).

### IOpr: Expansion, left-right asymmetry and neurochemistry

The IOpr expands in parallel to the cerebellum. This is reflected in the change in shape of the IOpr from a simple ribbon in rat and cat to an elongated ribbon with a very complex folding pattern in chimpanzee and human. Further, in chimpanzee and human, the IOpr folding pattern is different on the two sides of the brain. It is interesting to note that another structure, the dentate nucleus of the cerebellum, also undergoes a parallel increase in folding complexity (Sultan et al., 2010). While there have been many theories and studies of the mechanisms of cortical folding, the mechanisms of folding of these buried structures has not been analyzed.

Although the elaborate folding pattern of the IOpr was seen in both chimpanzee and the human, the variability among IOpr neurons in immunoreactivity to calcium-binding proteins was seen only in the human. Further, we have never seen the immunoreactivity to calcium-binding proteins in neurons in any other brainstem structure to vary among cases, in humans or in animals (Baizer and Baker, 2005, 2006a,b; Baizer et al., 2007, 2013a,b; Baizer and Broussard, 2010). These observations suggest unique characteristics of the neurochemistry of IOpr neurons in the human. Other evidence for human-specific neurochemical properties comes from studies showing a major accumulation of the age-related pigment lipofuscin in IOpr neurons (Mann and Yates, 1974; Mann et al., 1978; Drach et al., 1994, 1998; Ikeda et al., 1998; Gray and Woulfe, 2005). This pigment accumulation can affect cell function and protein synthesis, and may be a factor in age-related neurodegenerative disorders (Brunk and Terman, 2002; Moreno-Garcia et al., 2018). We also saw the accumulation of silver granules, typically associated with neurodegenerative disorders, in neurons of the IOpr of humans but not chimpanzees (Baizer et al., 2018).

The functional implications of the changes in the neurochemistry and the apparent loss of IOpr neurons are really not understood. It is possible that loss of climbing-fiber input to the cerebellum might be related to age-related changes in the ability to retain or learn motor skills, or even to the problems with balance and falls that are a major problem for the elderly.

### DC: Species differences in laminar organization

Auditory information leaves the cochlea in the eighth nerve. The first synapse is in the cochlear nuclei. The cochlear nuclei are divided into four subdivisions, the dorsal cochlear nucleus (DC), the anterior (VCP) and posterior (VCP) divisions of the ventral cochlear nucleus and the granule cell domain. In looking at the neuroanatomy of these structures it is important to keep in mind the differences in audition among species. Species differ in the frequency range detected, in head size, and in the size, shape and degree of mobility of the pinna (e.g., Heffner, 1997; Heffner and Heffner, 2007). The “classical” description of the DC, based on studies of the cat, is that is a laminar structure with a layer of pyramidal cells oriented perpendicular to the brainstem surface. Other studies, and our data, show that the degree of lamination varies among species, especially primates (Moore, 1980). In the human DC, the fusiform cells are oriented at all different angles relative to the brainstem surface. How this organization affects information processing in the human DC is unknown. It is also unclear how the variation in the DC organization among species underlies or reflects species differences in auditory function. Our data are consistent with the findings of species differences in the organization of other brainstem auditory nuclei, especially the medial nucleus of the trapezoid body (review in Kulesza and Grothe, 2015).

### The cerebral cortex and the human brainstem

In the brainstem, we found individual differences in the size and shape of all nuclei that we examined. In this respect, the brainstem echoes the cerebral cortex. Individual differences both in sulcal and gyral patterns, as well as the extent of different cytoarchitectonic areas are well-established for the human cerebral cortex (Seidenwurm et al., 1985; Paus et al., 1996; Bartley et al., 1997; review in Ochiai et al., 2004; Uylings et al., 2005; Caspers et al., 2006; Fornito et al., 2006; Cykowski et al., 2008; Scheperjans et al., 2008; Zlatkina and Petrides, 2010; Fedeli et al., 2020).

We also found left-right differences in the size and shape of the Arc and the folding pattern of the IOpr ribbon, with a unique pattern for each case. Structural and functional asymmetry are well established for several regions of the human cortex, but for cortex the direction of the left-right asymmetry is consistent across most cases. Anatomical asymmetry of the planum temporale and Sylvian fissure have been associated with functional asymmetry for language (Geschwind and Levitsky, 1968; Steinmetz et al., 1991; Witelson and Kigar, 1992; Foundas et al., 1999; Toga and Thompson, 2003; Dorsaint-Pierre et al., 2006). Structural asymmetry of the central sulcus (locus of motor cortex) and the postcentral gyrus (locus of somatosensory cortex) may be associated with hand preference (Amunts et al., 1996, 2000; Sörös et al., 1999; Hammond, 2002; Cykowski et al., 2008; Jung et al., 2008; Sun et al., 2012).

Both the IOpr and the Arc are precerebellar structures. The cerebellum clearly has a role in motor function, including control of the hand. There is evidence for functional asymmetry in the cerebellum related to hand preference (Snyder et al., 1995; Wang et al., 2013). We found qualitative left-right differences in the folding pattern of the IOpr. This suggested the hypothesis that there might be quantitative differences as well. We asked first if there was a greater extent or complexity of folding pattern of the IOpr ribbon contralateral to the preferred hand. We did not, however, see any left-right differences in those measures (Baizer et al., 2011). However, our analysis was limited. We did not compare size or number of neurons, cell spacing or packing density. Future quantitative analysis would be necessary to rule out the existence of left-right IOpr differences related to hand preference. A similar hypothesis might be proposed for the Arc, but we have not yet done any quantitative comparison of the composition of the Arc on left and right sides of the brain.

It is also possible that these precerebellar brainstem structures might be important for language or cognitive function. Anatomical studies showed that the cerebellum provides input via thalamic relays to cortical areas involved in cognitive function (Middleton and Strick, 1997, 2000; Dum et al., 2002). Those anatomical findings prompted many subsequent studies that have supported a role for the cerebellum in cognitive and language function (reviews in Mariën et al., 2014; Fiez, 2016). There is also evidence for functional asymmetry in the cerebellum related to language (Haberling and Corballis, 2016). It is therefore possible that the Arc or IOpr may have a role in mediating those functions.

### Approaches to studying structure and function in the human brain

Our analysis of brainstem structures has highlighted individual differences, with the goal of eventually correlating structural differences with functional correlates. There is a long history of studies of cerebral cortex whose goal was to determine localization of function. Early studies were case reports associating localized brain damage with loss of specific function, e.g., the discovery of the involvement of frontal cortex in speech, “Broca’s area” (review in Keller et al., 2009). There were then studies of groups of brain-damaged people in which specific regions were found to be associated with specific cognitive functions, for example the studies of Brenda Milner on the frontal lobes (review in Kolb, 2021). However, the overall perspective of these studies was that these relationships between structure and function would be universally applicable. Some studies of postmortem brains addressed individual differences in the relationship between variation in structure and variation in behavior: e.g., the correlation between brain volume and IQ measures (Witelson et al., 2006), the size of the corpus callosum and degree of right-hand preference (Witelson and Nowakowski, 1991), and the extent of cytoarchitectonic language areas in a “linguistic genius” (Amunts et al., 2004).

The development of structural and functional imaging has allowed the analysis of specific and complex functions in the living brain, and provided access to much larger groups of subjects than studies relying on brain-damaged subjects or postmortem brains. Some early studies addressed individual differences in assessing the relationship between structure and function, e.g., a study showing variation in the size of auditory cortex with pitch perception (Wong et al., 2008). However, the approach of many functional imaging studies has been to identify functional regions across individuals, for example the areas involved in face recognition (Haxby et al., 1996; Kanwisher et al., 1997; Gauthier et al., 2000; Zhen et al., 2013). However, those studies did not look for, or analyze, possible individual differences. The limitation of using the “standard” Talairach atlas which overrides differences among subjects in cortical anatomy has long been recognized (Rademacher et al., 1993; Uylings et al., 2005). Establishing the importance of considering individual differences in imaging studies, and how to do so, was a major goal of Gordon et al. (2017). More recent imaging studies increasingly acknowledge the importance of studying human individual differences (e.g., Seghier and Price, 2018; Hommelsen et al., 2022; Liegeois-Chauvel et al., 2022).

### Future directions in studies of the human brain: Genetic analysis and individual differences

There is much research on the genetic basis of brain development and function that implicitly recognizes the importance of individual differences. The studies fall into four major categories: (1) Normal brain development. Many studies are asking what are the genes critical for normal brain development, including patterns of cortical folding, the formation of sulci and gyri and of cortical asymmetry (e.g., Rubenstein and Rakic, 1999; Sun and Hevner, 2014; Mallela et al., 2020). It may be that subtle differences in gene expression among individuals accounts for some of the individual variability in brain structure that we see. (2) Genetic basis of variation in human skills. Another line of research seeks to identify critical genes associated with human skills that vary in the population, e.g., mathematical ability and music ability (some examples Tan et al., 2014; Warrier and Baron-Cohen, 2016; Henriksen et al., 2017). (3) Abnormal brain development. Other studies have identified gene mutations resulting in atypical brain development, some of which can result in developmental disorders such as autism spectrum disorders, Fragile X syndrome, and some seizure disorders like Dravet syndrome (Gleeson et al., 1998; Berkovic and Scheffer, 1999; Gaitanis and Walsh, 2004; Garber et al., 2008; Geschwind, 2011; Marini et al., 2011; Guerrini and Dobyns, 2014; Perucca et al., 2020). (4) Genes affecting risk of neurodegenerative and psychiatric disorders. Finally, there are studies to identify genes implicated in the vulnerability to adult-onset psychiatric (e.g., Sullivan, 2005; Li et al., 2018) or neurodegenerative disorders such as Parkinson’s disease, amyotrophic lateral sclerosis, fronto-temporal dementia or Alzheimer’s disease (e.g., Freischmidt et al., 2015; Giri et al., 2016; Lill, 2016; Bandres-Ciga et al., 2020; Sims et al., 2020). These disease-related genetic studies are of critical importance in developing recognition of the individual differences that are essential to the development of personalized or precision medicine.

All of these studies are generating a wealth of data that are likely to enhance our understanding of the development and functional implications of individual differences in brainstem structures.

## Data availability statement

The raw data supporting the conclusions of this article will be made available by the authors, without undue reservation.

## Ethics statement

Subjects and their next-of-kin provided written informed consent and the study was approved by the Research Ethics Board of McMaster University.

## Author contributions

JB conducted the experiments, analyzed the data, made the figures, and wrote the manuscript. SW assembled the Witelson Normal Brain Collection, supervised the dissection of the brains, and contributed to writing the manuscript. Both authors approved the submitted version.
